# Viral promoters can initiate expression of toxin genes introduced into *Escherichia coli*

**DOI:** 10.1186/1472-6750-5-19

**Published:** 2005-06-20

**Authors:** Astrid Lewin, Martin Mayer, Janet Chusainow, Daniela Jacob, Bernd Appel

**Affiliations:** 1 Robert Koch-Institut, Nordufer 20, 13353 Berlin, Germany; 2 HU-Berlin, Abt. Bakterienphysiologie, Chausseestr.117, 10115 Berlin, Germany; 3 Bioprocessing Technology Institute (BTI) 20 Biopolis Way, Centros Singapore 138668; 4 Bundesintitut für Risikobewertung, Diedersdorfer Weg 112277 Berlin, Germany

## Abstract

**Background:**

The expression of recombinant proteins in eukaryotic cells requires the fusion of the coding region to a promoter functional in the eukaryotic cell line. Viral promoters are very often used for this purpose. The preceding cloning procedures are usually performed in *Escherichia coli *and it is therefore of interest if the foreign promoter results in an expression of the gene in bacteria. In the case molecules toxic for humans are to be expressed, this knowledge is indispensable for the specification of safety measures.

**Results:**

We selected five frequently used viral promoters and quantified their activity in *E. coli *with a reporter system. Only the promoter from the thymidine kinase gene from HSV1 showed no activity, while the polyhedrin promoter from baculovirus, the early immediate CMV promoter, the early SV40 promoter and the 5' LTR promoter from HIV-1 directed gene expression in *E. coli*. The determination of transcription start sites in the immediate early CMV promoter and the polyhedrin promoter confirmed the existence of bacterial -10 and -35 consensus sequences. The importance of this heterologous gene expression for safety considerations was further supported by analysing fusions between the aforementioned promoters and a promoter-less cytotoxin gene.

**Conclusion:**

According to our results a high percentage of viral promoters have the ability of initiating gene expression in *E. coli*. The degree of such heterologous gene expression can be sufficient for the expression of toxin genes and must therefore be considered when defining safety measures for the handling of corresponding genetically modified organisms.

## Background

The production of recombinant proteins in eukaryotic cells requires the fusion of the target genes to promoter sequences functional in the eukaryotic cells and exhibiting the desired expression pattern. The molecular cloning procedures necessary for the construction of these fusions are usually performed in bacteria, mostly in *Escherichia coli *K12. A heterologous expression of powerful toxins like for example botulinum toxin, tetanus toxin or diphtheria toxin may pose a risk for the persons carrying out the experiments. A careful risk analysis considering an exposition to the toxin produced in the eukaryotic cells as well as a potential danger originating from toxin production by the recombinant *E. coli *has therefore to be carried out.

Since the nucleic acid sequences characterising eukaryotic-type and bacterial promoters are different, it is usually regarded improbable that promoter sequences functional in eukaryotic cells are able to direct a considerable transcription initiation in bacteria [1,2].

There are numerous differences between the transcription machinery of eukaryotes and eubacteria represented by different structures of promoters and the complexity of RNA polymerases and transcription factors [3-6]. On the other hand, the RNA polymerases are evolutionary conserved and belong to a protein family termed "multisubunit RNAP family" [4,7,8]. The subunits of the eubacterial RNA polymerase all have counterparts in the subunits of the RNA polymerase from Archaea and the three RNA polymerases from eukaryotes [4,8-11]. There is also an obvious similarity between the TATA box from eukaryotes and Archaea to the -10 consensus sequence from eubacterial promoters.

This discrepancy reflected by differences versus similarities of the transcription machinery of organisms belonging to different kingdoms motivated us to analyse the capacity of promoter sequences to direct gene expression in foreign hosts. In previous studies we have shown that a high percentage of eukaryotic-type promoters specific for plants can direct gene expression in eubacteria. By testing promoter activity of ten plant-specific promoters in five eubacterial species we could show that in 50 % of the tested combinations the promoter sequences could be used in the bacterial host [12,13]. In a subsequent study we demonstrated that not only eukaryotic promoter sequences but any type of eukaryotic DNA has a high probability to initiate transcription after transfer into bacteria. This was shown by transfer of random DNA fragments from the yeast *Saccharomyces cerevisiae *into *E. coli *K12 and detection of initiation of significant gene expression in about 80 % of the transformed *E. coli *[14].

In this study we addressed the question if heterologous gene expression in *E. coli *by viral promoters has to be considered when specifying safety measures for correspondent experiments. We selected five frequently used viral promoter sequences: (i) the polyhedrin promoter from baculovirus (P PH), (ii) the enhancer and immediate early promoter from CMV (P CMV), (iii) the early promoter from SV40 (P SV40), (iv) the thymidine kinase promoter from HSV1 (P TK), and (v) the 5'LTR promoter from HIV (P LTR). The heterologous gene expression of these promoters in *E. coli *K12 was tested using the *luxAB *genes from *Vibrio harveyi *as reporter system. Two of the promoters (P PH and P CMV) were analysed in more detail to determine the transcription start sites in *E. coli*. By additionally fusing these two promoters to a gene encoding a cytotoxin (hemolysin gene *vlly *from *Vibrio vulnificus*) the relevance of the heterologous gene expression by viral promoters for safety considerations was further supported.

## Results and discussion

### Lux gene expression in *E. coli *directed by viral promoters

**Figure 1:**
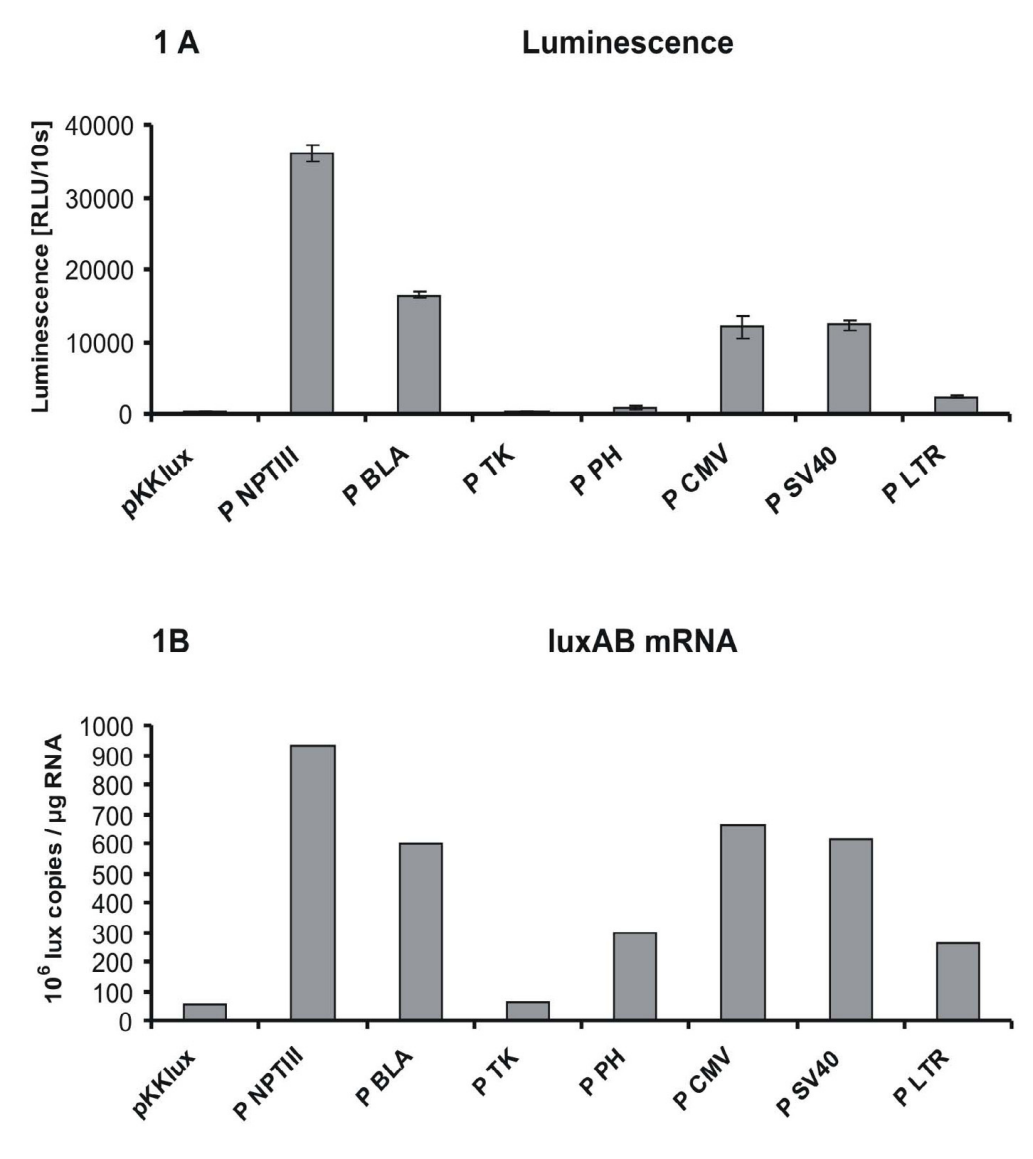
**Gene expression caused by viral promoters in *E. coli***. Luminescence (Panel A) and amount of lux AB mRNA transcripts determined by Real-time RT-PCR (Panel B) of cultures of *E. coli *containing fusions between the *luxAB *genes in the vector pKKlux and the viral promoters P TK (thymidine kinase promoter from HSV1), P PH (polyhedrin promoter from baculovirus), P CMV (immediate early promoter from CMV), P SV40 (early promoter from SV40) and P LTR (5' LTR promoter from HIV1) or the bacterial promoters P BLA (promoter from the TEM-1 β-lactamase gene) and P NPTIII (promoter from the neomycinphosphotransferase III gene). *E. coli *containing pKKlux was included in both experiments as negative control. The luminescence (Panel A) was measured in triplicate and the columns represent the average of the three measurements with the standard deviation. The lux AB mRNA (Panel B) was measured in duplicate and the columns represent the average of the two measurements.

To investigate whether viral promoters can initiate gene expression in bacteria, we constructed fusions between various viral promoters and the promoter-less *luxAB *genes from *V. harveyi*, transformed the fusions into *E. coli *K12 and measured the luminescence of the transformants. The viral promoters analysed were the polyhedrin promoter from baculovirus (P PH), the enhancer and immediate early promoter from CMV (P CMV), the early promoter from SV40 (P SV40), the thymidine kinase promoter from HSV1 (P TK), and the 5'LTR promoter from HIV (P LTR). As positive controls gene expression levels caused by homologous bacterial promoters in *E. coli *were determined by fusing the promoter from the *nptIII *gene (P NPTIII) and the promoter from the TEM-1 β-lactamase gene (P BLA) to the *luxAB *genes. The vector pKKlux without any promoter sequence was included as negative control. The results of the luminescence measurements are shown in Fig. 1A. The vector pKKlux generated a background luminescence (212 RLU/10 s), which was identical to the luminescence caused by the P TK construct (210 RLU/10 S). The P PH (824 RLU/10 s) initiated a weak gene expression in *E. coli*, while the P LTR (2382 RLU/10 s) caused a luminescence 10 times above background. The P CMV (11981 RLU/10 s) and the P SV40 (12301 RLU/10 s) initiated a strong gene expression in *E. coli*, which was comparable to the gene expression of the bacterial P BLA (16350 RLU/10s). The bacterial P NPTIII caused the highest luminescence values with 36011 RLU/10 s. In summary, four of the five viral promoters tested generated a luminescence at least four times above background (P PH, P CMV, P SV40, P LTR). The luminescence caused by the P CMV and P SV40 was even similar to the luminescence caused by the bacterial P BLA supporting the relevance of the heterologous gene expression with respect to the phenotypic expression of transferred traits.

Confirmation that our reporter assay reliably reflects the strength of the tested promoters was achieved by performing quantitative RT-PCR assays using primers and probes specific for the *luxB *gene (Fig. 1B).

### Mapping of DNA sequences in viral promoters serving as transcription start sites in *E. coli*

We chose two different approaches to determine the location of DNA sequences in the viral promoters which may be used as transcription initiation sites by the RNA polymerase from *E. coli*.

The first approach consisted in a statistical analysis of the nucleotide sequences of the promoters with a neural network promoter prediction program [15]. The number of predicted prokaryotic promoters in the five sequences analysed varied from zero to six. Surprisingly, the promoter prediction program identified no potential promoter sequence in the P SV40, which was the promoter causing the strongest luminescence in our reporter assays (Fig. 1A). The construct with the P CMV, which was almost as strong as the construct with the P SV40 in the luminescence assay, was shown by statistical analysis to have six potential start sites. In each of the remaining three promoter sequences (P PH, P TK, P LTR), the promoter prediction program predicted one potential start site. Interestingly, a potential promoter was also identified in the P TK sequence, although the P TK *luxAB *fusion pointed out to be negative in the luminescence assay.

In summary, it can be concluded that a prediction of the gene expression activity of a DNA sequence solely on the basis of a statistical analysis of the sequence is not reliable, and that only an experimental approach using a reporter system can provide this information. This result is in good agreement with the outcome of our previous studies [12-14] on the functionality of plant promoters in bacteria and on the promoter activity of yeast DNA in *E. coli*. There we also demonstrated that it was not possible to reliably predict the occurrence of a bacterial promoter in a DNA sequence by statistical analysis.

Our second approach consisted in the experimental determination of transcription start sites by the 5' RACE-method and sequencing of the 5' RACE products. For this purpose, we selected two viral promoters which had both been shown to direct gene expression according to the results of the luminescence measurements. The construct with the P CMV belonged to the viral promoters initiating a very strong heterologous gene expression, while the P PH caused a weak heterologous gene expression in *E. coli*. Constructs containing the bacterial promotersP BLA and P NPIII were included into the experiments as positive controls.

**Figure 2:**
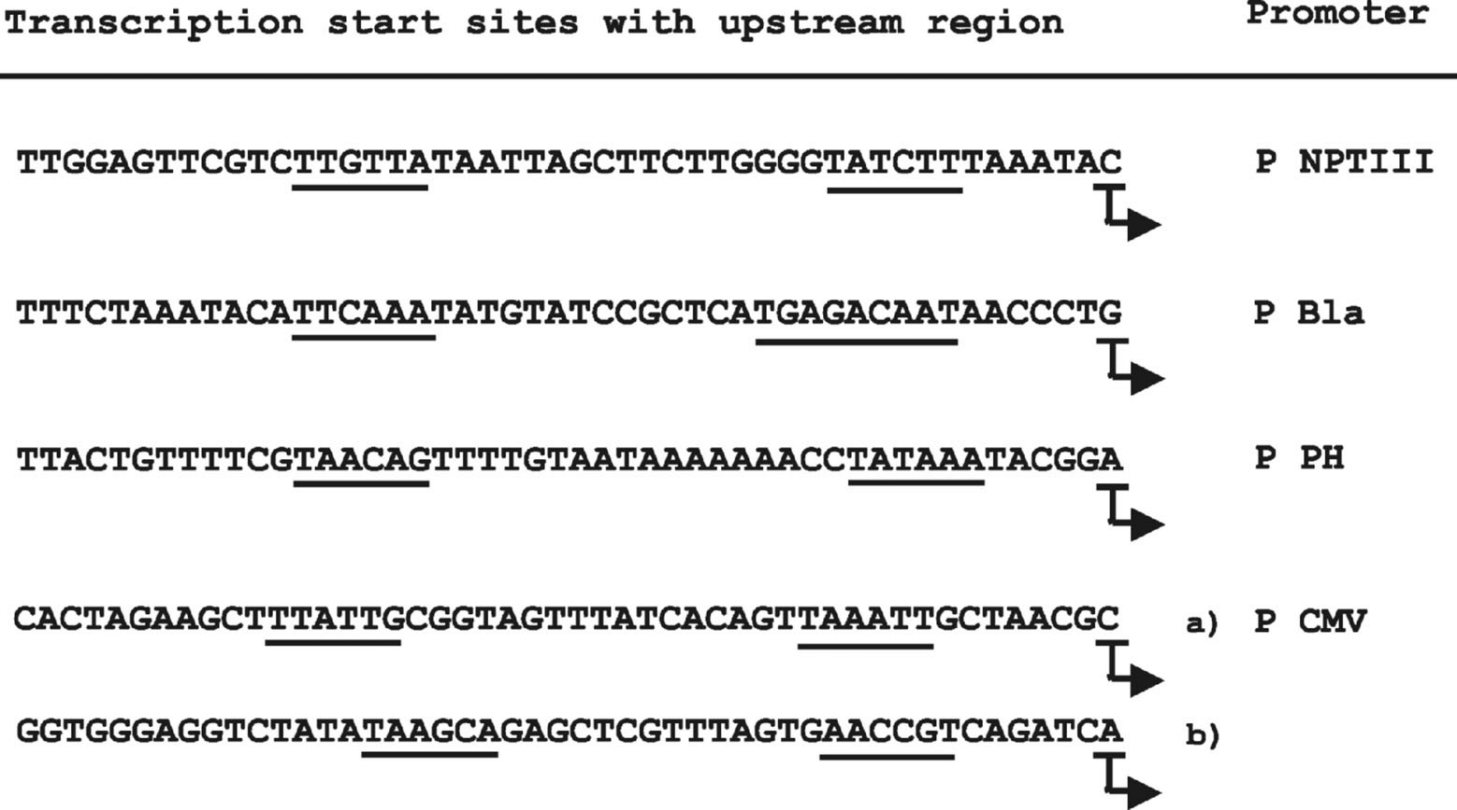
**Identification of transcription start sites**. Transcription start sites were identified by the 5' RACE method in *E. coli *containing pKKlux derivatives with fusions between the promoterless luxAB genes and the promoter from the *nptIII *gene (P NPTIII), the promoter from the TEM-1 gene (P BLA), the polyhedrin promoter (P PH) and the immediate early CMV promoter (P CMV). The sequences shown cover the transcription initiation sites identified in *E. coli *(indicated by arrows) and their upstream regions. The putative -10 and -35 regions are underlined. Two transcription start sites [a), b)] were identified in the P CMV.

The results summarised in Fig. 2 show the transcription start sites and the location of the putative -10 and -35-regions. The transcription start sites determined in the P BLA and P NPTIII promoter regions by the 5' RACE method were identical with the start sites described by other authors [16,17] confirming our experimental approach.

We could identify specific start sites in the two tested constructs containing the viral promoters P PH and P CMV confirming that the luminescence resulted from specific transcription initiation events. One start site was identified in the P PH, whereas two start sites were found in the P CMV (Fig. 2). In the upstream region of all transcription start sites we found at a distance of 5 to 7 bp a region with similarity to the -10 (TATAAT) consensus sequence which was separated by 14 to 18 bp from a region with similarity to the -35 (TTGACA) consensus sequence.

It was very astonishing, that none of the transcription start sites within the viral promoters determined by the 5' RACE method corresponded to the transcription initiation sites predicted by the promoter prediction programme (data not shown). The difficulties encountered in the efforts to predict promoter activities in bacteria by statistical analysis can at least in part be explained by the importance of the nucleotide sequences surrounding the -10 and -35 regions which determine the physical-chemical and structural characteristics of the DNA and the promoter strength [18,19]. Jacquet and Reiss [18], for example, analysed the influence of the context of the -10 and -35 regions on transcription efficiencies and found that transcription efficiencies varied by a factor of ten depending on the sequences surrounding the consensus sequences. Progress in bacterial promoter prediction has been made using neural network programs like the one we have employed for our statistical analysis. Demeler and Zhou [20] reported a prediction accuracy of 98.4 % using a neural network for the prediction of *E. coli *promoters. Further problems of promoter predictions consist in the existence of different sigma factors binding to different recognition sequences and in the variation between the consensus sequences of different bacterial species.

### Expression of the hemolysin gene *vlly *from *V. vulnificus *in *E. coli *directed by viral promoters

As heterologous gene expression was observed using luciferase as reporter system, we were interested, if the promoter activity of the viral promoter sequences in *E. coli *was sufficiently strong to significantly express virulence factors. This would be of importance for risk assessments of corresponding genetically modified organisms. For instance it has been shown that the transfer of the inv locus necessary for the invasion of *Yersinia pseudotuberculosis *into host cells enabled a non-invasive strain of *E. coli *to penetrate cultured cells [21]. Similar observations have been made with the invasion locus of *Mycobacterium avium*. After transfer of this locus into non-invasive *E. coli *and *Mycobacterium smegmatis *the recipients could invade epithelial cells [22]. A worst case scenario would be the heterologous expression of a toxin able to operate without being dependent on other virulence factors. Many hemolysin genes fulfil this condition. The phenotypic effect of hemolysins can be relatively easily monitored and hemolysin genes are prevalent in many pathogenic bacteria. We selected the *vlly *gene from *V. vulnificus *as reporter gene [23]. An advantage of *vlly *for our purposes is its small size (1071 bp) facilitating the amplification and cloning procedures. Furthermore, a 1.3 kb fragment of *V. vulnificus *DNA carrying *vlly *has been shown to confer a hemolytic phenotype onto *E. coli *[23].

We inserted the promoter-less *vlly *(including the Shine-Dalgarno-Sequence) downstream from the viral promoters P PH and P CMV present in the pKKlux constructs and transformed the promoter-*vlly *fusions into *E. coli *K12. As negative control, *vlly *was inserted into pKKlux without any promoter sequences.

**Figure 3:**
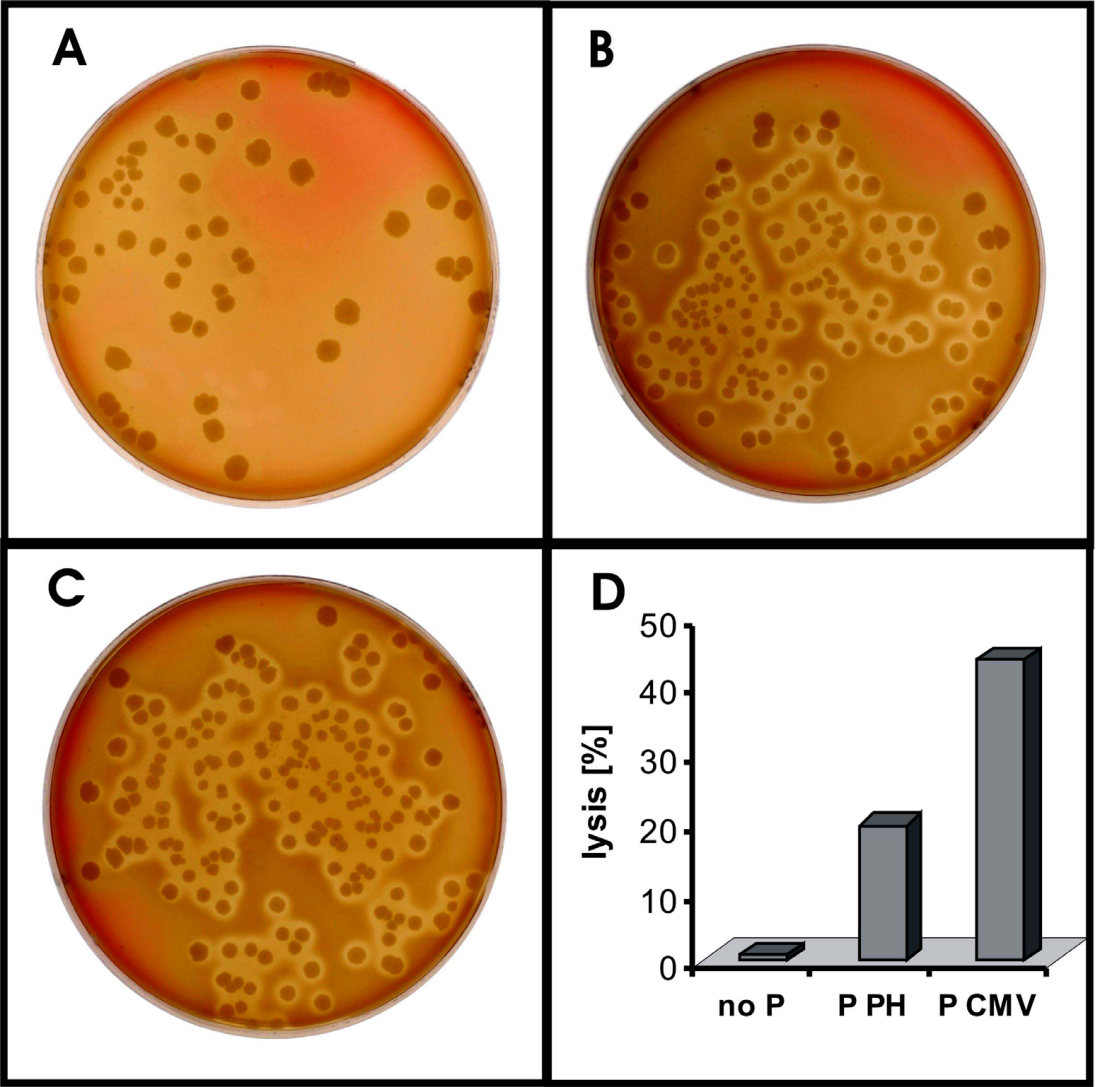
**Expression of hemolysin genes in *E. coli *directed by viral promoters**. Hemolysis of sheep blood erythrocytes by *E. coli *DH5α containing the *vlly *coding sequence without promoter (panels A and D), with the promoter from the polyhedrin gene (P PH: panels B and D) or with the immediate early promoter from CMV (P CMV: panels C and D). Panels A to C show the hemolysis in blood agar plates visible as cleared zones around the colonies. Panel D indicates the percentage of erythrocytes lysed in a liquid blood assay. The columns show the average of two measurements.

We first monitored the *vlly *expression by plating the transformants onto blood agar plates and observing hemolysis zones around the colonies. *E. coli *containing P PH as well as *E. coli *containing P CMV were hemolytic (Fig. 3B,C). However, a quantification to what extent a lysis of erythrocytes had occurred, was not possible with this method.

We therefore also measured release of haemoglobin from lysed erythrocytes in a liquid hemolysis assay. Since Vlly had been shown before to be located in the periplasma and cytoplasma of recombinant *E. coli *[23], our assay involved sonication of the *E. coli *cells to liberate periplasmic and cytoplasmic Vlly. The outcome of a typical hemolysis assay is shown in Fig. 3D. Both viral promoters initiated an expression of *vlly*. In accordance with the luminescence assays, the P CMV directed a very strong *vlly *expression resulting in lysis of almost half the erythrocytes present in the assay, while the P PH was much weaker causing a lysis of about 20% of erythrocytes.

## Conclusion

We showed that four (polyhedrin gene promoter from baculovirus, immediate early promoter from CMV, early promoter from SV40 and LTR promoter from HIV 1) out of five tested viral promoters carry structural features required by the bacterial RNA polymerase to initiate transcription. Two promoters (P CMV and P SV40) were as strong as the promoter from the bacterial TEM1-β-lactamase gene. The determination of the transcription start sites in selected viral promoters confirmed the presence of sequences with homology to the bacterial -10 and -35 promoter consensus sequences. Two promoters (P CMV and P PH) were shown to be able to direct expression of a bacterial cytotoxin, which illustrated the relevance of this type of heterologous gene expression for the specification of safety measures for the handling of genetically modified organisms. A strong expression of a foreign gene by a heterologous promoter must either be taken into consideration when defining safety measures or if necessary, it must be excluded by performing appropriate experiments. If there is the need for exclusion of a heterologous gene expression, care should be taken in choosing convenient promoter sequences. Alternatively, site-directed mutagenesis can be employed to design promoter sequences according to the experimental requirements. By exchanging nucleotides, which are required for binding of the bacterial RNA-polymerase, but which are of no or little importance for the transcription initiation in the final eukaryotic recipient, it is possible to minimise the heterologous gene expression while maintaining the desired characteristics of the promoter with regard to its function in eukaryotic cells or tissues [13]. Finally, the generation of a functional gene product not only depends on the presence of functional promoter sequences but can be also influenced by factors like the presence or absence of introns, the codon usage, post-translational modifications or the necessity of protein secretion.

## Methods

### Bacteria and growth conditions

*Escherichia coli *K12 strain DH5α [24] and *Vibrio vulnificus *strain CH1603 (O:8, isolated from the Baltic Sea, Germany) were grown at 37°C overnight in LB medium [25].

### Measurement of luminescence

The measurement of luciferase activity was performed as described before [12]. Bacterial cultures were grown at 28°C up to an optical density (λ = 600 nm) of 1.0 to 1.3 and diluted to contain 10^6 ^cells per ml. After transfer of 100 μl (microliter) of the diluted cultures into microtiter plates, 50 μl of 2 % decanal in 50 mM sodium phosphate buffer, pH 7.0, were added and the luminescence (RLU: relative light units) was measured in triplicate at 28°C for 10 s in the Microlumat LB96P from EG&G Berthold (Bad Wildbad, Germany).

### Detection of hemolysis

To detect hemolysis on blood agar plates, a broth culture of the bacteria was either streaked or plated onto enterohemolysin agar plates (Oxoid, Wesel, Germany) and incubated at 37°C. The plates were evaluated visually after 40 hours.

A quantification of the hemolytic activity of the bacteria was achieved using defibrinated sheep blood (Oxoid, Wesel, Germany) that was washed three times with PBS buffer (137 mM NaCl, 2.7 mM KCl, 10 mM Na_2_HPO_4_, 2 mM KH_2_PO_4_, pH 7.4). The bacteria were incubated at 37°C up to an optical densitiy (λ = 588 nm) of 1.3. To liberate intracellular hemolysin, the cells were disrupted using ultrasound for 1 min at 50 watt in the Branson Sonifier 450 (Branson Ultrasonics Corporation, Danbury, CT, USA). The disrupted cell lysate was at once placed on ice and centrifuged for 10 min at 9300 g. 970 μl of the supernatant were spiked with 20 μl washed blood and 10 μl 1 M CaCl_2 _and incubated at 37°C for 45 min. Every 5 min the suspension was carefully mixed. The mixture was then centrifuged at 1500 g for 10 min and the haemoglobin in the supernatant was quantified by measuring the OD at 540 nm. The value for complete lysis of erythrocytes (OD>2) was obtained by using distilled water instead of culture supernatant. All cultures were measured in duplicate.

### Molecular biology techniques

Common molecular biology techniques (DNA isolation, restriction digestion, ligation, electrophoresis) were carried out according to standard protocols [25] or according to the recommendations of the manufacturers of kits and enzymes. Sequencing reactions were performed by using the Prism Big Dye™ FS Terminator Cycle Sequencing Ready Reaction Kit from PE Applied Biosystems, Weitersheim, Germany. Transformation of *E. coli *was performed according to the method of Hanahan [24].

### Construction of recombinant plasmids

Viral promoters as well as bacterial promoters were inserted into the vector pKKlux [12]. pKKlux (7.7 kb) is a derivative of the promoter probe vector pKK232-8 [26], which carries a promoter-less *cat *(chloramphenicol-acetyltransferase) gene in front of the multiple cloning site. Read-through into the *cat *gene is prevented by the transcription terminator of the *rrn*b gene from *E. coli. *pKK232-8 contains a beta-lactamase gene to allow selection by adding ampicillin (100 μg/ml) into the medium. pKKlux was generated by inserting in front of the *cat *gene the promoterless luciferase genes *luxAB *from *Vibrio harveyi *present in plasmid pUT/mini-Tn5 *lux*AB [27], allowing the determination of promoter activities by measuring luminescence.

The promoter fragments were amplified by PCR using primers provided with restriction sites for the restriction enzymes *Sma*I and *Xba*I allowing ligation into the *Sma*I / *Xba*I sites of pKKlux. This cloning strategy guaranteed correct orientation of the promoters upstream from the promoter-less *luxAB *genes. Only the PTK, which contains a *Sma*I site, was not digested with *Sma*I prior to ligation of the PCR fragment into pKKlux.

Table 1 describes the generated promoter fragments and the templates and primers used for their amplification.

For cloning of the hemolysin gene *vlly *from *V. vulnificus *[23,28], a 1118 bp fragment carrying a promoterless *vlly *was amplified using the primers vlly-S (GG*TCTAGA*GCAGTCTAAAAGGAGAAAGTCATGGTGG) and vlly-AS (GG*TCTAGA*CCCGATGAGGAAAGGTGATCC). The primers had been provided with *Xba*I restriction sites allowing insertion of *vlly *in the *Xba*I site of the pKK232-8-derivatives containing the promoters described in Table 1. Correct orientation of *vlly *downstream of the analysed promoters was confirmed by sequencing.

### Quantification of lux mRNA

Bacterial cultures were grown over night in LB medium at 28°C. 200 μl of these cultures were inoculated into 5 ml of LB medium and grown for 4 to 6 hours to an optical density (λ = 600 nm) of 1.0. Aliquots of these cultures were taken for RNA isolation. RNA was isolated with the SV Total RNA Isolation System Kit from Promega, Madison, USA, which includes a DNase digestion step. The RT-PCR was performed with the Titan™ One Tube RT-PCR System from Boehringer Mannheim (Indianapolis, Ind., USA) in the ABI Prism 7700 Sequence Detection system (Applied BioSystems Division, Perkin Elmer, Foster City, Calif., USA). The primer pair used for the quantitative RT-PCR [Lux2270F (CCGTTAACCCACACGCGT) and Lux2329R (TGCTCGTCGCATTCACAAA)] amplified a 60 bp fragment from the *luxB *gene. The dually labelled detector probe hybridising within the sequence between these primers had the sequence (FAM)-CACTGAAGGCGGTCCTGCGCA-(TAMRA). The RT-PCR was performed with 0.1 μg RNA in 50 μl reaction mix according to the recommendations of the manufacturer (with RT-reaction buffer, 1.5 mM MgCl_2_, 0.2 mM of each dNTP, 1 μM downstream primer, 1 μM upstream primer, 5 mM DTT, 5 U RNase inhibitor, 1 μl of enzyme mix containing AMV, Taq DNA polymerase and Pwo DNA polymerase). The quantitative TaqMan RT-PCR furthermore required addition of 180 nM dually labelled probe and 1 μM ROX (6-Carboxy-X-rhodamin) as passive reference dye. The reverse transcription was carried out at 50°C for 30 min and terminated by heating at 95°C for 10 min. Amplification was carried out by running 35 cycles with 30 sec at 95°C and 1 min at 60°C. Samples were measured in duplicate. The amount of the lux mRNA was determined by the help of a standard established with known amounts (0.073 pg to 730 pg) of the plasmid pKKlux.

### Mapping of transcription start sites with the 5' RACE method

Total RNA from broth cultures was isolated with the SV Total RNA Isolation System Kit from Promega, Madison, USA. The 5' ends of the lux transcripts were mapped with the 5' RACE (Rapid Amplification of cDNA Ends) System Kit from Life Technologies, Inc., Rockville, USA. 1 to 5 μg of total RNA was used to synthesise cDNA from the 5' end of the lux mRNA with the gene-specific primer GSP1/luxA (CAACATAAGGATTCCC). The reaction was performed with the SuperScript II RT at 42°C for 50 min. A homopolymeric C-tail was added to the cDNA with the terminal deoxynucleotidyl transferase. The RACE products were synthesised using the abridged anchor primer GGCCACGCGTCGACTATACGGGIIGGGIIGGGIIG and the gene-specific primer GSP2/luxA (GCGTACTAGTCAGTGAAGTGGTGCTCTAGCAACC). The PCR program was run with 35 cycles composed of a denaturation step of 1 min at 94°C, an annealing step of 30 sec at 65°C and an elongation step of 2 min at 68°C. The RACE products were directly sequenced to identify transcription start sites.

## Authors' contributions

AL: design of project, preparation of the manuscript, statistical analysis of nucleotide sequences for promoter search. MM: construction of recombinant plasmids, luminescence measurements, measurement of hemolysis, real-time PCR, determination of transcription start sites. JC: construction of recombinant plasmids, luminescence measurements, real-time PCR, determination of transcription start sites. DJ: preparation of and instructions to 5'RACE. BA: conception of project and follow-up discussions of results. All authors read and approved the final manuscript.

## Figures and Tables

**Table 1:** Promoters analysed in this study. The promoters (column 1 and 4) were amplified by PCR using the template DNA (column 2) and the primers (column 3) listed in the table.

Promoter fragment	Template DNA^a^	Primer sequence^b^	Size of promoter-fragment
**P PH**: Polyhedrin promoter from baculovirus	pBacPAK8 [29]	PH-1: G*CCCGGG*CCATCTCGCAAATAAATAAG PH-2: G*TCTAGA*CAGGGATCCGTATTTATAGG	78 bp
**P CMV**: Enhancer and immediate early promoter from CMV	pRL-CMV [30]	CMV-1: GC*CCGGG*GATCTTCAATATTGGCCATTAGCC CMV-2: G*TCTAGA*CACTGACTGCGTTAGCAATTTAAC	802 bp
**P SV40**: Early promoter from SV40	pGL3 [31]	SV40-1: G*CCCGGG*CTAGCCCGGGCTCGAGATCTG SV40-2: G*TCTAGA*TTTGCAAAAGCCTAGGCCTCC	223 bp
**P TK **Thymidine kinase promoter from HSV1	pRL-TK [32]	TK-1: G*CCCGGG*ATCTAAATGAGTCTTCGGACCTCGC TK-2: TCTAGACGAGACTGTTGTGTCAGAAGAATCAAGC	788 bp
**P LTR **HIV-1 subtype D 5'LTR promoter	isolate 92UG021	LTR-1: GG*CCCGG*GATTAGATATCCACTGACCTTTGGATGGTGC LTR-2: GG*TCTAGA*TTAAGCAGTGGGTTCCCTAGCTAGCC	412 bp
**P NPTIII**: *nptIII *promoter	pBin19 [33, 34]	NPTIII-S: GCG*CCCGGG*CATAATTGTGGTTTCAAAATCGGC NPTIII-AS: GG*GTCTAG*ATTATTATTTCCTTCCTCTTTTC	193 bp
**P BLA**: TEM-1 promoter	pKK232-8 [26, 35]	Amp-S: GCG*CCCGGG*CGTCAGGTGGCACTTTTCG Amp-AS: GG*GTCTAG*AACTCTTCCTTTTTCAATATTATTG	134 bp

^a^pBacPAK8 was from Clontech (Palo Alto, CA, USA), pRL-CMV, pGL3 and pRL-TK were from Promega (Madison, WI, USA). The 5'LTR promoter sequence was kindly provided by S. Somogyi.

^b^Restriction sites added to the primers for cloning purposes are italicised.
